# Synthesis and anticancer activity of novel quinazolinone-based rhodanines

**DOI:** 10.1186/s13065-017-0333-x

**Published:** 2017-10-13

**Authors:** Sherihan El-Sayed, Kamel Metwally, Abdalla A. El-Shanawani, Lobna M. Abdel-Aziz, Harris Pratsinis, Dimitris Kletsas

**Affiliations:** 1 Department of Medicinal Chemistry, Faculty of Pharmacy, Zagazig University, Zagazig, Egypt; 2 Laboratory of Cell Proliferation and Ageing, Institute of Biosciences and Applications, National Centre of Scientific Research “Demokritos”, Athens, Greece

**Keywords:** Rhodanines, Anticancer, Apoptosis, Reactive oxygen species

## Abstract

**Background:**

Rhodanines and quinazolinones have been reported to possess various pharmacological activities.

**Results:**

A novel series of twenty quinazolinone-based rhodanines were synthesized via Knoevenagel condensation between 4-[3-(substitutedphenyl)-3,4-dihydro-4-oxoquinazolin-2-yl)methoxy]substituted-benzaldehydes and rhodanine. Elemental and spectral analysis were used to confirm structures of the newly synthesized compounds. The newly synthesized compounds were biologically evaluated for in vitro cytotoxic activity against the human fibrosarcoma cell line HT-1080 as a preliminary screen using the MTT assay.

**Conclusions:**

All the target compounds were active, displaying IC_50_ values roughly in the range of 10–60 µM. Structure–activity relationship study revealed that bulky, hydrophobic, and electron withdrawing substituents at the *para*-position of the quinazolinone 3-phenyl ring as well as methoxy substitution on the central benzene ring, enhance cytotoxic activity. The four most cytotoxic compounds namely, **45**, **43**, **47**, and **37** were further tested against two human leukemia cell lines namely, HL-60 and K-562 and showed cytotoxic activity in the low micromolar range with compound **45** being the most active, having IC_50_ values of 1.2 and 1.5 μM, respectively. Interestingly, all four compounds were devoid of cytotoxicity against normal human fibroblasts strain AG01523, indicating that the synthesized rhodanines may be selectively toxic against cancer cells. Mechanistic studies revealed that the most cytotoxic target compounds exhibit pro-apoptotic activity and trigger oxidative stress in cancer cells.
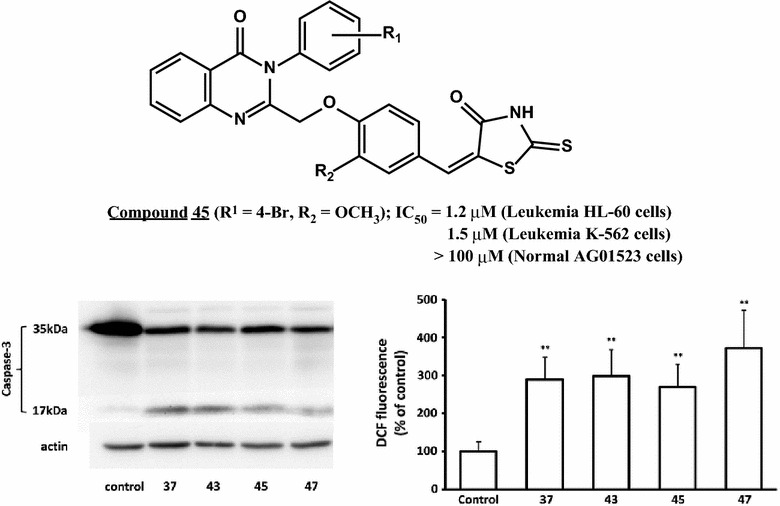

## Introduction

Cancer is still one of the leading causes of death worldwide and the pursuit of novel clinically useful anticancer agents is therefore, one of the top priorities for medicinal chemists. Although gaining a reputation in recent years as “frequent hitters” in screening programs, rhodanines as well as their bioisosteres, 2,4-thiazolidinediones and the hydantoins, remain attractive tools to medicinal chemists for structural manipulations directed at developing potent and selective ligands for a wide array of potential molecular targets. There has been a growing debate in the medicinal chemistry community in the last few years about the usefulness of rhodanines and related compounds as scaffolds or templates in drug discovery and drug development. In a recent comparative study on the rhodanines and related heterocycles, it was concluded that such scaffolds can serve as attractive building blocks rather than being promiscuous binders or multi-target chemotypes [1]. In the drug market, epalrestat is a rhodanineacetic acid derivative marketed in Japan since 1992 for the treatment of diabetic peripheral neuropathy. It acts by inhibiting aldose reductase which is the key enzyme in the polyol pathway of glucose metabolism under hyperglycemic conditions. Epalrestat was reported to be generally well tolerated on long-term use and it causes only few adverse effects such as nausea, vomiting and elevation of liver enzyme levels [2–7]. From a positive perspective, the good clinical safety profile of epalrestat justified our interest in rhodanines as potential therapeutic candidates. Literature survey revealed extensive research work on the anticancer effects of rhodanines over the last few decades [8–30]. On the molecular level, rhodanines were found to induce apoptosis through modulation of the pro-survival proteins of the Bcl-2 family [8–12] or through modulation of other key signaling proteins [13–16]. Interestingly, reactive oxygen species (ROS) have been reported to be up-regulated after rhodanine treatment, a fact possibly associated with mitochondria-mediated apoptosis [14, 29, 30]. Rhodanines were also reported to exert their anticancer effects through inhibition of phosphatase of regenerating liver (PRL-3) [16, 17]. On the other hand, numerous reports of quinazolinones as anticancer agents have appeared in literature [31–35]. Based on these findings, we were interested in investigating the anticancer effects of this novel scaffold of quinazolinone-based rhodanines, being isosteric to our previously reported 2,4-thiazolidindediones. In the present investigation, a series of twenty quinazolinone-based rhodanines were synthesized and tested for in vitro cytotoxic activity against the human fibrosarcoma cell line HT-1080 using the MTT assay. The four most active compounds namely, **45**, **43**, **47**, and **37** were selected for further testing against two human leukemia cell lines (HL-60 and K-562) and the normal human fibroblasts strain AG01523, and their mechanism of action was investigated.

## Results and discussion

### Chemistry

**Scheme 1:**
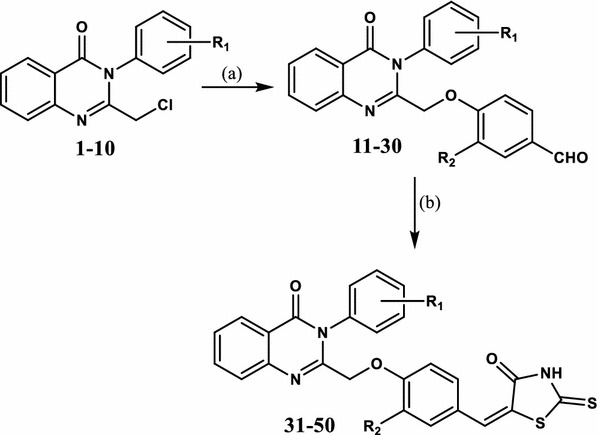
Reagents and conditions: **a** 4-hydroxybenzaldehyde or vanillin, K_2_CO_3_, KI, acetonitrile, reflux, 3 h. **b** Rhodanine, sodium acetate, glacial acetic acid, reflux, 24–48 h

A straight forward synthetic pathway was adopted to synthesize the target compounds **31**–**50** as depicted in Scheme 1. The intermediate chloromethylquinazolinones (**1**–**10**) were prepared following reported procedures from anthranilic acid in two steps [36–39]. The *N*-chloroacetylation step was effected through reaction of anthranilic acid with chloroacetyl chloride in dry benzene under reflux conditions. The cyclization step was achieved by condensing the *N*-chloroacetyl derivatives with the appropriate anilines in presence of phosphorous oxychloride in dry toluene. Reaction of chloromethylquinazolinones (**1**–**10**) with 4-hydroxybenzaldehyde or vanillin under the basic conditions of potassium carbonate in the presence of potassium iodide to catalyze the alkylation, afforded the aldehyde derivatives (**11**–**30**) in good yields as previously reported by us [40]. Finally, the desired title rhodanines (**31**–**50**) were obtained by treatment of the aldehydes with rhodanine under Knoevenagel condensation conditions using sodium acetate as a catalyst. The target compounds were structurally characterized by means of ^1^H NMR and ^13^C NMR spectrometric methods. Characteristically, the rhodanine NH proton appeared at 13.75–13.77 ppm as a broad singlet. The azomethine proton appeared within the aromatic region as a sharp singlet around 7.57 ppm. In ^13^C NMR spectra, the thiocarbonyl carbon appeared in the range of 195–196 ppm. Compounds having trifluoromethyl groups namely, **37** and **47**, showed two characteristic quartets due to C–F coupling. Other aliphatic and aromatic carbons appeared at their expected chemical shifts. The purity of the target compounds was satisfactorily confirmed by elemental analysis.

### Biological study

The target compounds were initially screened for their in vitro cytotoxic activities against the human fibrosarcoma cell line HT-1080 using the MTT assay. As shown in Table 1, all compounds were active, and their IC_50_ values were roughly in the region between 10 and 60 μM. Close inspection of biological data of the tested compounds led to several observations on their structure–activity relationships. The best cytotoxic activity was displayed by compounds bearing a bulky, hydrophobic, and electron-withdrawing substituent at the *para*-position of the quinazolinone 3-phenyl ring as evidenced by the relatively low IC_50_ values of compounds **45** (R_1_ = 4-Br, R_2_ = OCH_3_; IC_50_ = 8.7 µM), **43** (R_1_ = 4-Cl, R_2_ = OCH_3_; IC_50_ = 10.2 µM), **47** (R_1_ = 4-CF_3_, R_2_ = OCH_3_; IC_50_ = 15.8 µM), and **37** (R_1_ = 4-CF_3_, R_2_ = H; IC_50_ = 15.8 µM). As a general pattern, *meta*-substituted compounds were found less active as compared to their *para*-substituted counterparts. Moreover, methoxy substitution on the central benzene ring appears to enhance cytotoxicity as evidenced by the lower IC_50_ values of compounds **41**–**50** in comparison to their unsubstituted analogues **31**–**40**. The four most cytotoxic compounds were selected for further testing, starting with their cytotoxicity against two human leukemia cell lines (HL-60 and K-562) and the normal human skin fibroblast strain AG01523. As shown in Table 2, the leukemia cells were more sensitive to all four compounds, compared to HT-1080 cells, and compound **45** was again the most active compound, with IC_50_ values 1.2 and 1.5 μM, for HL-60 and K-562 cells, respectively. Other compounds tested displayed three to fourfold lower activity against the two cell lines tested. Interestingly, normal human fibroblasts were not affected by all four compounds, indicating that the synthesized rhodanines may be selectively toxic against cancer cells.

**Table 1:** Cytotoxicity of test compounds against HT-1080 cells


Code	R_1_	R_2_	HT-1080
**31**	H	H	42.9 (± 17.3)
**32**	4-F	H	47.3 (± 0.3)
**33**	4-Cl	H	35.3 (± 4.6)
**34**	3-Cl	H	57.1 (± 9.2)
**35**	4-Br	H	28.8 (± 2.4)
**36**	3-Br	H	43.4 (± 6.7)
**37**	4-CF_3_	H	15.8 (± 2.1)
**38**	4-CH_3_	H	35.7 (± 5.9)
**39**	3-CH_3_	H	47.5 (± 12.1)
**40**	4-OCH_3_	H	36.4 (± 0.9)
**41**	H	OCH_3_	36.1 (± 2.9)
**42**	4-F	OCH_3_	35.8 (± 11.8)
**43**	4-Cl	OCH_3_	10.2 (± 4.7)
**44**	3-Cl	OCH_3_	28.4 (± 9.4)
**45**	4-Br	OCH_3_	8.7 (± 3.6)
**46**	3-Br	OCH_3_	23.7 (± 0.6)
**47**	4-CF_3_	OCH_3_	15.8 (± 2.1)
**48**	4-CH_3_	OCH_3_	31.5 (± 4.1)
**49**	3-CH_3_	OCH_3_	30.7 (± 1.1)
**50**	4-OCH_3_	OCH_3_	34.7 (± 2.7)
Doxorubicin	–	–	0.012 (± 0.005)

IC_50_s (μM), mean of three independent experiments (± standard deviation)

**Table 2:** Cytotoxicity of selected compounds against a panel of cell strains


Code	R_1_	R_2_	Cell line		
			HL-60	K-562	AG01523
**37**	4-CF_3_	H	5.5 (± 0.2)	5.0 (± 3.2)	> 100
**43**	4-Cl	OCH_3_	5.1 (± 2.0)	4.5 (± 4.3)	> 100
**45**	4-Br	OCH_3_	1.2 (± 0.5)	1.5 (± 0.2)	> 100
**47**	4-CF_3_	OCH_3_	2.6 (± 0.4)	5.1 (± 0.3)	> 100
Doxorubicin	–	–	0.011 (± 0.006)	0.212 (± 0.074)	0.875 (± 0.248)

IC_50_s (μM), mean of three independent experiments (± standard deviation)

**Fig. 1:**
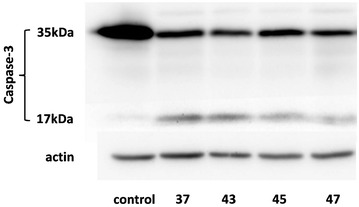
Apoptosis of HL-60 cells following rhodanine treatment. Cells incubated with the indicated compounds (50 μM) or the corresponding concentration of vehicle (control) for 48 h were lysed, and caspase-3 cleavage was monitored by Western analysis of cell lysates (one representative experiment out of two similar ones is depicted)

**Fig. 2:**
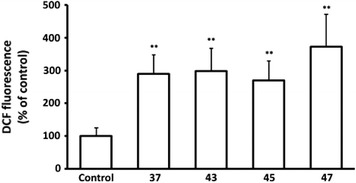
Oxidative stress of HT-1080 cells following rhodanine treatment. Cells were incubated with the indicated compounds (10 μM) or the corresponding concentration of vehicle (control). Intracellular ROS were determined after 48 h using the DCFH-DA method. Results represent the mean ± standard deviation of three independent experiments (**p < 0.01)

Regarding the mechanistic aspects of the above cytotoxic activity, flow cytometric analysis of DNA content did not reveal significant changes in the cell cycle phase distribution of rhodanine-treated HT-1080 cells compared with control ones, with the exception of an S-phase arrest caused by compounds **43** and **45** at 48 h (not shown). All four compounds were found to induce apoptosis of HL-60 cells, based on caspase-3 cleavage (Fig. 1), in accordance with the numerous literature reports [8–16, 29, 30]. Furthermore, all four compounds were found to significantly induce intracellular ROS accumulation in HT-1080 cells following a 48-h treatment (Fig. 2), in agreement with similar observations in other cancer cell lines using different rhodanine molecules [29, 30].

## Experimental

### Chemistry

#### General

Melting points are uncorrected and were measured on a Gallenkamp melting point apparatus. ^1^H and ^13^C NMR spectra were recorded on Bruker 400-MHz, JEOL RESONANCE 500-MHz, and Varian-Mercury 300-MHz spectrometers. Chemical shifts were expressed in parts per million (ppm) downfield from tetramethylsilane (TMS) and coupling constants (*J*) were reported in Hertz. Elemental analyses (C, H, N) were performed at the Microanalytical Unit, Cairo university, and the Regional Center for Mycology and Biotechnology, Al-Azhar University, Cairo, Egypt. All compounds were routinely checked by thin-layer chromatography (TLC) on aluminum-backed silica gel plates. Flash column chromatography was performed using silica gel (100–200 mesh) with the indicated solvents. All solvents used in this study were dried by standard methods. The starting 2-(chloromethyl)-3-(substitutedphenyl)quinazolin-4(3H)-ones (**1**–**10**) [36–39] and 4-[3-(substitutedphenyl)-3,4-dihydro-4-oxoquinazolin-2-yl)methoxy]substitutedbenzaldehydes (**11**–**30**) [40] were synthesized following reported procedures.

#### Synthetic procedures

##### General procedure for the synthesis of 5-{4-[(3-substitutedphenyl-4-oxo-3,4-dihydroquinazolin-2-yl)methoxy]substitutedbenzylidene}-2-thioxothiazolidin-4-ones **31**–**50**

A mixture of the appropriate aldehyde (10 mmol), rhodanine (20 mmol), and sodium acetate (20 mmol) in glacial acetic acid (10 ml), was heated under reflux for 48 h. After cooling to room temperature, the reaction mixture was poured into water and the precipitate was filtered, washed with water and dried. The crude product was subjected to silica gel column chromatography using methylene chloride/methanol (99:1) as an eluent followed by recrystallization from DMF/EtOH or DMF/H_2_O.

###### 5-{4-[(3-Phenyl-4-oxo-3,4-dihydroquinazolin-2-yl)methoxy]benzylidene}-2-thioxothiazolidin-4-one (**31**)

Yield: 54%, mp 230–232 °C (DMF/EtOH); ^1^H NMR (DMSO-d_6_, ppm): δ 4.83 (s, 2H, CH_2_), 6.99–7.03 (m, 2H, Ar–H), 7.43–7.62 (m, 9H, Ar–H, azomethine-H), 7.70–7.72 (d, *J* = 800 Hz, 1H, Ar–H), 7.86–7.88 (m, 1H, Ar–H), 8.15–8.17 (dd, *J*_*1*_ = 1.20 Hz, *J*_*2*_ = 8.00 Hz, 1H, Ar–H), 13.75 (s, 1H, NH). ^13^C NMR (DMSO-d_6_, ppm): δ 67.75 (CH_2_), 115.61 (2C), 121.06, 122.64, 126.05, 126.42, 127.37, 127.57, 128.71 (2C), 129.19, 129.28 (2C), 131.59, 132.42 (2C), 134.83, 135.88, 146.66, 151.07, 159.45, 161.16, 169.41, 195.48 (C=S). Anal. calcd for C_25_H_17_N_3_O_3_S_2_: C, 63.68; H, 3.63; N, 8.91. Found: C, 63.55; H, 3.88; N, 8.60.

###### 5-{4-[(3-(4-Fluorophenyl)-4-oxo-3,4-dihydroquinazolin-2-yl)methoxy]benzylidene}-2-thioxothiazolidin-4-one (**32**)

Yield: 46%, mp 231–233 °C, dec. (DMF/H_2_O); ^1^H NMR (DMSO-d_6_, ppm): δ 4.86 (s, 2H, CH_2_), 7.02–7.04 (d, *J* = 8.80 Hz, 2H, Ar–H), 7.33–7.38 (m, 2H, Ar–H), 7.50–7.52 (d, *J* = 8.80 Hz, 2H, Ar–H), 7.59 (s, 1H, azomethine-H), 7.61–7.65 (m, 3H, Ar–H), 7.71–7.73 (d, *J* = 8.00 Hz, 1H, Ar–H), 7.87–7.91 (m, 1H, Ar–H), 8.16–8.18 (d, *J* = 7.60 Hz, 1H, Ar–H), 13.76 (s, 1H, NH). ^13^C NMR (DMSO-d_6_, ppm): δ 68.28 (CH_2_), 116.13 (2C), 116.55, 116.78, 121.49, 123.17, 126.58, 126.92, 127.86, 128.12, 131.49 (d, *J* = 9.0 Hz), 132.09, 132.52, 132.92 (2C), 135.39, 147.10, 151.59, 159.91, 161.30, 161.78, 163.75, 169.92, 195.97 (C = S). Anal. calcd for C_25_H_16_FN_3_O_3_S_2_: C, 61.34; H, 3.29; N, 8.58. Found: C, 61.27; H, 3.63; N, 8.51.

###### 5-{4-[(3-(4-Chlorophenyl)-4-oxo-3,4-dihydroquinazolin-2-yl)methoxy]benzylidene}-2-thioxothiazolidin-4-one (**33**)

Yield: 51%, mp 121–123 °C (DMF/H_2_O); ^1^H NMR (DMSO-d_6_, ppm): δ 4.88 (s, 2H, CH_2_), 7.01–7.03 (d, *J* = 8.00 Hz, 2H, Ar–H), 7.48–7.50 (d, *J* = 8.00 Hz, 2H, Ar–H), 7.57–7.58 (m, 6H, Ar–H, azomethine-H), 7.70–7.72 (d, *J* = 8.00 Hz, 1H, Ar–H), 7.86–7.90 (m, 1H, Ar–H), 8.15–8.17 (d, *J* = 7.60 Hz, 1H, Ar–H), 13.76 (s, 1H, NH). ^13^C NMR (DMSO-d_6_, ppm): δ 68.28 (CH_2_), 116.14 (2C), 121.46, 123.19, 126.61, 126.93, 127.88, 128.16, 129.84 (2C), 131.22 (2C), 132.09, 132.91 (2C), 134.35, 135.29, 135.43, 147.08, 151.35, 159.87, 161.66, 169.92, 195.98 (C=S). Anal. calcd for C_25_H_16_ClN_3_O_3_S_2_: C, 59.34; H, 3.19; N, 8.30. Found: C, 58.90; H, 3.59; N, 8.29.

###### 5-{4-[(3-(3-Chlorophenyl)-4-oxo-3,4-dihydroquinazolin-2-yl)methoxy]benzylidene}-2-thioxothiazolidin-4-one (**34**)

Yield: 55%, mp 237–239 °C (DMF/H_2_O); ^1^H NMR (DMSO-d_6_, ppm): δ 4.89 (s, 2H, CH_2_), 7.01–7.03 (m, 2H, Ar–H), 7.49–7.64 (m, 7H, Ar–H, azomethine-H), 7.72–7.75 (m, 2H, Ar–H), 7.88–7.92 (m, 1H, Ar–H), 8.16–8.18 (dd, *J*_*1*_ = 1.20 Hz, *J*_*2*_ = 8.00 Hz, 1H, Ar–H), 13.76 (s, 1H, NH). ^13^C NMR (DMSO-d_6_, ppm): δ 67.87 (CH_2_), 115.60 (2C), 121.00, 122.75, 126.14, 126.43, 127.41, 127.69 (2C), 129.09, 129.28, 130.75, 131.54, 132.39 (2C), 133.34, 134.94, 137.24, 146.57, 150.69, 159.34, 161.10, 169.47, 195.49 (C=S). Anal. Calcd for C_25_H_16_ClN_3_O_3_S_2_: C, 59.34; H, 3.19; N, 8.30. Found: C, 59.47; H, 3.44; N, 8.22.

###### 5-{4-[(3-(4-Bromophenyl)-4-oxo-3,4-dihydroquinazolin-2-yl)methoxy]benzylidene}-2-thioxothiazolidin-4-one (**35**)

Yield: 52%, mp 171–174 °C (DMF/H_2_O); ^1^H NMR (DMSO-d_6_, ppm): δ 4.88 (s, 2H, CH_2_), 7.01–7.03 (d, *J* = 8.40 Hz, 2H, Ar–H), 7.49–7.54 (m, 4H, Ar–H), 7.58–7.62 (m, 2H, Ar–H, azomethine-H), 7.70–7.72 (d, *J* = 8.40 Hz, 3H, Ar–H), 7.86–7.90 (t, *J* = 7.60 Hz, 1H, Ar–H), 8.14–8.16 (d, *J* = 8.00 Hz, 1H, Ar–H), 13.75 (s, 1H, NH). ^13^C NMR (DMSO-d_6_, ppm): δ 68.27 (CH_2_), 116.17 (2C), 121.47, 122.97, 123.16, 126.61, 126.92, 127.89, 128.13, 131.52 (2C), 132.11, 132.80 (2C), 132.91 (2C), 135.41, 135.76, 147.09, 151.30, 159.89, 161.60, 169.89, 195.96 (C=S). Anal. calcd for C_25_H_16_BrN_3_O_3_S_2_: C, 54.55; H, 2.93; N, 7.63. Found: C, 54.19; H, 3.20; N, 7.49.

###### 5-{4-[(3-(3-Bromophenyl)-4-oxo-3,4-dihydroquinazolin-2-yl)methoxy]benzylidene}-2-thioxothiazolidin-4-one (**36**)

Yield: 48%, mp 252–254 °C (DMF/H_2_O); ^1^H NMR (DMSO-d_6_, ppm): δ 4.88 (s, 2H, CH_2_), 7.00–7.02 (d, *J* = 8.80 Hz, 2H, Ar–H), 7.43–7.51 (m, 3H, Ar–H), 7.57–7.64 (m, 4H, Ar–H, azomethine-H), 7.71–7.73 (d, *J* = 8.00 Hz, 1H, Ar–H), 7.86–7.91 (m, 2H, Ar–H), 8.15–8.17 (d, *J* = 8.00 Hz, 1H, Ar–H), 13.75 (s, 1H, NH). ^13^C NMR (DMSO-d_6_, ppm): δ 68.41 (CH_2_), 116.10 (2C), 121.51, 122.05, 123.37, 126.67, 126.93, 127.91, 128.20, 128.53, 131.50, 131.95, 132.36, 132.64, 132.87 (2C), 135.44, 137.85, 147.06, 151.19, 159.80, 161.62, 170.11, 196.07 (C=S). Anal. Calcd for C_25_H_16_BrN_3_O_3_S_2_: C, 54.55; H, 2.93; N, 7.63. Found: C, 54.72; H, 3.08; N, 7.53.

###### 5-{4-[(3-(4-Trifluoromethylphenyl)-4-oxo-3,4-dihydroquinazolin-2-yl)methoxy]benzylidene}-2-thioxothiazolidin-4-one (**37**)

Yield: 38%, mp 153–155 °C (DMF/H_2_O); ^1^H NMR (DMSO-d_6_, ppm): δ 4.89 (s, 2H, CH_2_), 6.96–6.99 (d, *J* = 8.80 Hz, 2H, Ar–H), 7.47–7.49 (d, *J* = 8.80 Hz, 2H, Ar–H), 7.57 (s, 1H, azomethine-H), 7.61–7.65 (m, 1H, Ar–H), 7.72–7.81 (m, 3H, Ar–H), 7.90–7.93 (m, 2H, Ar–H), 8.04 (s, 1H, Ar–H), 8.17–8.18 (d, *J* = 7.20 Hz, 1H, Ar–H), 13.76 (s, 1H, NH). ^13^C NMR (DMSO-d_6_, ppm): δ 68.56 (CH_2_), 115.92 (2C), 121.52, 122.79, 123.24, 125.50, 126.47 (partially resolved q, *J* = 4.0 Hz), 126.65, 126.93, 127. 93, 128.28, 130.31 (q, *J* = 32.0 Hz), 130.96, 132.03, 132.84 (2C), 133.60, 135.50, 137.28, 147.06, 151.13, 159.65, 161.76, 169.88, 195.96 (C=S). Anal. calcd for C_26_H_16_F_3_N_3_O_3_S_2_: C, 57.88; H, 2.99; N, 7.79. Found: C, 57.82; H, 3.22; N, 7.73.

###### 5-{4-[(3-(4-Methylphenyl)-4-oxo-3,4-dihydroquinazolin-2-yl)methoxy]benzylidene}-2-thioxothiazolidin-4-one (**38**)

Yield: 51%, mp 148–150 °C (DMF/H_2_O); ^1^H NMR (DMSO-d_6_, ppm): δ 2.08 (s, 3H, CH_3_), 4.84 (s, 2H, CH_2_), 6.99–7.02 (d, *J* = 8.70 Hz, 2H, Ar–H), 7.29–7.32 (d, *J* = 7.80 Hz, 2H, Ar–H), 7.37–7.42 (m, 2H, Ar–H), 7.47–7.50 (d, *J* = 8.70 Hz, 2H, Ar–H), 7.56–7.61 (m, 2H, Ar–H, azomethine-H), 7.67–7.70 (d, *J* = 8.10 Hz, 1H, Ar–H), 7.83–7.88 (m, 1H, Ar–H), 8.14–8.16 (d, *J* = 8.10 Hz, 1H, Ar–H), 13.75 (s, 1H, NH). ^13^C NMR (DMSO-d_6_, ppm): δ 20.68 (CH_3_), 67.62 (CH_2_), 115.66 (2C), 120.48, 121.01, 122.61, 126.01, 126.38, 127.31, 128.36 (2C), 129.78 (2C), 131.57, 132.38 (2C), 133.20, 134.73, 138.68, 146.63, 151.24, 159.52, 161.84, 169.36, 195.44 (C=S). Anal. Calcd for C_26_H_19_N_3_O_3_S_2_: C, 64.31; H, 3.94; N, 8.65. Found: C, 63.98; H, 3.71; N, 8.80.

###### 5-{4-[(3-(3-Methylphenyl)-4-oxo-3,4-dihydroquinazolin-2yl)methoxy]benzylidene}-2-thioxothiazolidin-4-one (**39**)

Yield: 54%, mp 165–168 °C (DMF/EtOH); ^1^H NMR (DMSO-d_6_, ppm): δ 2.31 (s, 3H, CH_3_), 4.83 (s, 2H, CH_2_), 6.99–7.01 (d, *J* = 8.80 Hz, 2H, Ar–H), 7.23–7.25 (d, *J* = 7.60 Hz, 1H, Ar–H), 7.31–7.39 (m, 3H, Ar–H), 7.48–7.50 (d, *J* = 8.80 Hz, 2H, Ar–H), 7.57 (s, 1H, azomethine-H), 7.60–7.62 (d, *J* = 8.00 Hz, 1H, Ar–H), 7.70–7.72 (d, *J* = 8.00 Hz, 1H, Ar–H), 7.85–7.89 (m, 1H, Ar–H), 8.14–8.16 (dd, *J*_*1*_ = 1.20 Hz, *J*_*2*_ = 8.00 Hz 1H, Ar–H), 13.75 (s, 1H, NH). ^13^C NMR (DMSO-d_6_, ppm): δ 20.73 (CH_3_), 67.79 (CH_2_), 115.60 (2C), 121.05, 122.73, 125.55, 126.06, 126.40, 127.37, 127.58, 129.05, 129.21, 129.77, 131.54, 132.39 (2C), 134.81, 135.73, 138.85, 146.64, 151.12, 159.47, 161.14, 169.53, 195.54 (C=S). Anal. Calcd for C_26_H_19_N_3_O_3_S_2_: C, 64.31; H, 3.94; N, 8.65. Found: C, 64.00; H, 3.87; N, 8.87.

###### 5-{4-[(3-(4-Methoxyphenyl)-4-oxo-3,4-dihydroquinazolin-2-yl)methoxy]benzylidene}-2-thioxothiazolidin-4-one (**40**)

Yield: 50%, mp 190–192 °C, dec. (DMF/EtOH); ^1^H NMR (DMSO-d_6_, ppm): δ 3.78 (s, 3H, OCH_3_), 4.85 (s, 2H, CH_2_), 7.03–7.06 (dd, *J*_*1*_ = 3.20 Hz *J*_*2*_ = 8.80 Hz, 4H, Ar–H), 7.45–7.47 (d, *J* = 8.00 Hz, 2H, Ar–H), 7.50–7.52 (d, *J* = 8.80 Hz, 2H, Ar–H), 7.57–7.61 (m, 2H, Ar–H, azomethine-H), 7.68–7.70 (d, *J* = 8.00 Hz, 1H, Ar–H), 7.85–7.89 (t, *J* = 7.20 Hz, 1H, Ar–H), 8.15–8.17 (d, *J* = 7.20 Hz, 1H, Ar–H), 13.76 (s, 1H, NH). ^13^C NMR (DMSO-d_6_, ppm): δ 55.34 (OCH_3_), 67.66 (CH_2_), 114.46 (2C), 115.68 (2C), 120.41, 121.03, 122.60, 126.01, 126.38, 127.29, 127.42, 128.23, 129.78 (2C), 131.59, 132.39 (2C), 134.70, 146.64, 151.54, 159.44, 161.34, 169.36, 195.44 (C=S). Anal. calcd for C_26_H_19_N_3_O_4_S_2_: C, 62.26; H, 3.82; N, 8.38. Found: C, 62.33; H, 3.92; N, 8.18.

###### 5-{4-[(3-Phenyl-4-oxo-3,4-dihydroquinazolin-2-yl)methoxy]-3-methoxybenzylidene}-2-thioxothiazolidin-4-one (**41**)

Yield: 50%, mp 243–246 °C (DMF/H_2_O); ^1^H NMR (DMSO-d_6_, ppm): δ 3.83 (s, 3H, OCH_3_), 4.79 (s, 2H, CH_2_), 6.94–6.96 (d, *J* = 8.40 Hz, 1H, Ar–H), 7.03–7.06 (dd, *J*_*1*_ = 1.60 Hz, *J*_*2*_ = 8.40 Hz, 1H, Ar–H), 7.16 (m, 1H, Ar–H), 7.36–7.62 (m, 7H, Ar–H, azomethine-H), 7.70–7.72 (d, *J* = 8.00 Hz, 1H, Ar–H), 7.86–7.90 (t, *J* = 7.60 Hz, 1H, Ar–H), 8.15–8.17 (d, *J* = 8.00 Hz, 1H, Ar–H). ^13^C NMR (DMSO-d_6_, ppm): δ 55.69 (OCH_3_), 68.35 (CH_2_), 113.87, 113.94, 121.05, 122.86, 124.02, 126.41, 126.53, 127.39, 127.59, 128.77, 129.15 (2C), 131.95 (3C), 134.84, 135.79, 146.66, 149.19, 149.25, 151.12, 161.17, 169.36, 195.44 (C=S). Anal. calcd for C_26_H_19_N_3_O_4_S_2_: C, 62.26; H, 3.82; N, 8.38. Found: C, 62.17; H, 3.80; N, 8.12.

###### 5-{4-[(3-(4-Fluorophenyl)-4-oxo-3,4-dihydroquinazolin-2-yl)methoxy]-3-methoxybenzylidene}-2-thioxothiazolidin-4-one (**42**)

Yield: 49%, mp 163–166 °C (DMF/EtOH); ^1^H NMR (DMSO-d_6_, ppm): δ 3.84 (s, 3H, OCH_3_), 4.83 (s, 2H, CH_2_), 6.99–7.01 (d, *J* = 8.40 Hz, 1H, Ar–H), 7.06–7.08 (d, *J* = 8.40 Hz, 1H, Ar–H), 7.17 (s, 1H, Ar–H), 7.31–7.35 (m, 2H, Ar–H), 7.58–7.63 (m, 4H, Ar–H, azomethine-H), 7.71–7.73 (d, *J* = 8.40 Hz, 1H, Ar–H), 7.87–7.91 (m, 1H, Ar–H), 8.16–8.18 (d, *J* = 7.60 Hz, 1H, Ar–H), 13.77 (s, 1H, NH). ^13^C NMR (DMSO-d_6_, ppm): δ 56.16 (OCH_3_), 68.90 (CH_2_), 114.29, 114.40, 116.40, 116.63, 121.49, 123.39, 124.50, 126.92, 127.06, 127.89, 128.14, 131.53, 131.62, 132.44, 135.39, 147.09, 149.65 (2C), 151.64, 161.30, 161.79, 163.75, 169.87, 195.94 (C=S). Anal. calcd for C_26_H_18_FN_3_O_4_S_2_: C, 60.11; H, 3.49; N, 8.09. Found: C, 59.91; H, 3.59; N, 7.80.

###### 5-{4-[(3-(4-Chlorophenyl)-4-oxo-3,4-dihydroquinazolin-2-yl)methoxy]-3-methoxybenzylidene}-2-thioxothiazolidin-4-one (**43**)

Yield: 52%, mp 239–241 °C (DMF/H_2_O); ^1^H NMR (DMSO-d_6_, ppm): δ 3.82 (s, 3H, OCH_3_), 4.84 (s, 2H, CH_2_), 6.99–7.07(m, 2H, Ar–H), 7.16 (s, 1H, Ar–H), 7.55–7.63 (m, 6H, Ar–H, azomethine-H), 7.70–7.72 (d, *J* = 8.00 Hz, 1H, Ar–H), 7.87–7.90 (t, *J* = 7.20 Hz, 1H, Ar–H), 8.15–8.17 (d, *J* = 8.00 Hz, 1H, Ar–H), 13.76 (s, 1H, NH). ^13^C NMR (DMF-d_7_, ppm): δ 56.19 (OCH_3_), 69.56 (CH_2_), 114.59, 114.68, 122.05, 124.37, 124.63, 127.17, 127.79, 128.30 (2C), 130.01 (2C), 131.76 (2C), 132.30, 134.98, 135.47, 135.99, 147.71, 150.22, 150.40, 151.97, 162.15, 170.63, 196.76 (C=S). Anal. calcd for C_26_H_18_ClN_3_O_4_S_2_: C, 58.26; H, 3.38; N, 7.84. Found: C, 58.52; H, 3.34; N, 7.99.

###### 5-{4-[(3-(3-Chlorophenyl)-4-oxo-3,4-dihydroquinazolin-2-yl)methoxy]-3-methoxybenzylidene]-2-thioxothiazolidin-4-one (**44**)

Yield: 52%, mp 147–150 °C (DMF/EtOH); ^1^H NMR (DMF-d_7_, ppm): δ 3.94 (s, 3H, OCH_3_), 4.99 (s, 2H, CH_2_), 7.09–7.10 (d, *J* = 8.50 Hz, 1H, Ar–H), 7.14–7.16 (d, *J* = 8.00 Hz, 1H, Ar–H), 7.23 (s, 1H, Ar–H), 7.50–7.56 (m, 2H, Ar–H), 7.57 (s, 1H, azomethine-H), 7.61–7.67 (m, 2H, Ar–H), 7.71–7.73 (d, *J* = 8.00, 1H, Ar–H), 7.82 (s, 1H, Ar–H), 7.89–7.92 (t, 1H, Ar–H), 8.18–8.20 (d, *J* = 7.00 Hz, 1H, Ar–H). ^13^C NMR (DMF-d_7_, ppm): δ 56.22 (OCH_3_), 69.60 (CH_2_), 114.43, 114.45, 122.03, 124.19, 124.58, 127.15, 127.71, 128.30, 128.36, 128.61, 129.96, 130.25, 131.27, 132.38, 134.42, 135.48, 138.40, 147.62, 150.11, 150.30, 151.76, 162.09, 170.39, 196.60 (C=S). Anal. calcd for C_26_H_18_ClN_3_O_4_S_2_: C, 58.26; H, 3.38; N, 7.84. Found: C, 57.97; H, 3.47; N, 7.88.

###### 5-{4-[(3-(4-Bromophenyl)-4-oxo-3,4-dihydroquinazolin-2-yl)methoxy]-3-methoxybenzylidene}-2-thioxothiazolidin-4-one (**45**)

Yield: 51%, mp 214–217 °C (DMF/H_2_O) ^1^H NMR (DMSO-d_6_, ppm): δ 3.80 (s, 3H, OCH_3_), 4.85 (s, 2H, CH_2_), 6.98–7.16 (m, 2H, Ar–H), 7.46–7.72 (complex m, 8H, Ar–H, azomethine-H), 7.85–7.91 (m, 1H, Ar–H), 8.14–8.17 (dd, *J*_*1*_ = 1.20 Hz, *J*_*2*_ = 8.10 Hz, 1H, Ar–H). ^13^C NMR (DMSO-d_6_, ppm): δ 55.66 (OCH_3_), 68.43 (CH_2_), 113.84, 114.03, 120.40, 120.94, 122.38, 122.90, 123.96, 126.40, 127.38, 127.64, 131.00 (2C), 131.94, 132.12 (2C), 134.89, 135.14, 146.56, 149.10, 149.18, 150.86, 161.08, 169.31, 195.41 (C=S). Anal. calcd for C_26_H_18_BrN_3_O_4_S_2_: C, 53.80; H, 3.13; N, 7.24. Found: C, 53.85; H, 2.79; N, 7.18.

###### 5-{4-[(3-(3-Bromophenyl)-4-oxo-3,4-dihydroquinazolin-2-yl)methoxy]-3-methoxybenzylidene}-2-thioxothiazolidin-4-one (**46**)

Yield: 42%, mp 243–245 °C (DMF/H_2_O); ^1^H NMR (DMSO-d_6_, ppm): δ 3.85 (s, 3H, OCH_3_), 4.83 (s, 2H, CH_2_), 6.99–7.07 (m, 2H, Ar–H), 7.16 (d, *J* = 1.60 Hz, 1H, Ar–H), 7.41–7.45 (t, *J* = 8.00 Hz, 1H, Ar–H), 7.55–7.64 (m, 4H, Ar–H, azomethine H), 7.73–7.75 (d, *J* = 8 Hz, 1H, Ar–H), 7.79 (s, 1H, Ar–H), 7.88–7.92 (t, *J* = 7.60 Hz, 1H, Ar–H), 8.15–8.17 (d, *J* = 8.00 Hz, 1H, Ar–H), 13.76 (s, 1H, NH). ^13^C NMR (DMSO-d_6_, ppm): δ 56.24 (OCH_3_), 69.01 (CH_2_), 114.15, 114.17, 121.50, 121.94, 123.36, 124.49, 126.94, 127.06, 127.94, 128.27, 128.55, 131.34, 132.41, 132.49, 132.59, 135.47, 137.74, 147.03, 149.50, 149.58, 151.23, 161.63, 169.83, 195.92 (C=S). Anal. calcd for C_26_H_18_BrN_3_O_4_S_2_: C, 53.80; H, 3.13; N, 7.24. Found: C, 53.50; H, 2.96; N, 7.21.

###### 5-{4-[(3-(4-(Trifluoromethylphenyl)-4-oxo-3,4-dihydroquinazolin-2yl)methoxy]-3-methoxybenzylidene}-2-thioxothiazolidin-4-one (**47**)

Yield: 43%, mp 165–168 °C (DMF/EtOH); ^1^H NMR (DMSO-d_6_, ppm): δ 3.79 (s, 3H, OCH_3_), 4.83 (s, 2H, CH_2_), 6.98–7.05 (m, 2H, Ar–H), 7.13 (s, 1H, Ar–H), 7.56 (s, 1H, azomethine-H), 7.61–7.65 (t, *J* = 7.60, 1H, Ar–H), 7.69–7.79 (m, 3H, Ar–H), 7.86–7.93 (m, 2H, Ar–H), 7.96 (s, 1H, Ar–H), 8.16–8.18 (d, *J* = 8.00 Hz, 1H, Ar–H), 13.76 (s, 1H, NH). ^13^C NMR (DMSO-d_6_, ppm): δ 56.00 (OCH_3_), 69.09 (CH_2_), 114.02 (2C), 121.52, 122.76, 123.40, 124.40, 125.47, 126.53 (partially resolved q, *J* = 4.0 Hz), 126.94, 127.09, 127.97, 128.34, 129.84 (q, *J* = 32.0 Hz), 130.83, 132.44, 133.71, 135.52, 137.13, 147.04, 149.32, 149.54, 151.19, 161.76, 169.82, 195.92 (C=S). Anal. calcd for C_27_H_18_F_3_N_3_O_4_S_2_: C, 56.94; H, 3.19; N, 7.38. Found: C, 56.63; H, 3.31; N, 7.49.

###### 5-{4-[(3-(4-Methylphenyl)-4-oxo-3,4-dihydroquinazolin-2-yl)methoxy]-3-methoxybenzylidene}-2-thioxothiazolidin-4-one (**48**)

Yield: 46%, mp 172–175 °C (DMF/H_2_O); ^1^H NMR (DMSO-d_6_, ppm): δ 2.32 (s, 3H, CH_3_), 3.83 (s, 3H, OCH_3_), 4.81 (s, 2H, CH_2_), 6.95–6.97 (d, *J* = 8.40, 1H, Ar–H), 7.04–7.06 (dd, *J*_*1*_ = 1.60 Hz, *J*_*2*_ = 8.40 Hz, 1H, Ar–H), 7.16–7.17 (d, *J* = 1.60 Hz, 1H, Ar–H), 7.28–7.30 (d, *J* = 8.40 Hz, 2H, Ar–H), 7.38–7.40 (d, *J* = 8.40 Hz, 2H, Ar–H), 7.57–7.61 (m, 2H, Ar–H, azomethine-H), 7.68–7.70 (d, *J* = 8.00 Hz, 1H, Ar–H), 7.84–7.89 (m, 1H, Ar–H), 8.14–8.16 (m, 1H, Ar–H), 13.76 (s, 1H, NH). ^13^C NMR (DMSO-d_6_, ppm): δ 21.20 (CH_3_), 56.18 (OCH_3_), 68.73 (CH_2_), 114.40, 114.49, 121.53, 123.32, 124.51, 126.91, 127.00, 127.86, 128.01, 128.92 (2C), 130.20 (2C), 132.47, 133.65, 135.27, 139.17, 147.16, 149.71, 149.82, 151.83, 161.72, 169.85, 195.93 (C=S). Anal. calcd for C_27_H_21_N_3_O_4_S_2_: C, 62.90; H, 4.11; N, 8.15. Found: C, 62.96; H, 4.17; N, 7.98.

###### 5-{4-[(3-(3-Methylphenyl)-4-oxo-3,4-dihydroquinazolin-2-yl)methoxy]-3-methoxybenzylidene}-2-thioxothiazolidin-4-one (**49**)

Yield: 47%, mp 154–156 °C (DMF/H_2_O); ^1^H NMR (DMSO-d_6_, ppm): δ 2.27 (s, 3H, CH_3_), 3.84 (s, 3H, OCH_3_), 4.79 (s, 2H, CH_2_), 6.94–6.96(d, *J* = 8.40 Hz, 1H, Ar–H), 7.04–7.06 (d, *J* = 8.40 Hz, 1H, Ar–H), 7.17–7.37 (m, 5H, Ar–H), 7.57–7.62 (m, 2H, Ar–H, azomethine-H), 7.71–7.73 (d, *J* = 8.00 Hz, 1H, Ar–H), 7.87–7.90 (t, *J* = 7.60 Hz, 1H, Ar–H), 8.15–8.17 (d, *J* = 7.60 Hz, 1H, Ar–H), 13.76 (s, 1H, NH). ^13^C NMR (DMSO-d_6_, ppm): δ 21.18 (CH_3_), 56.18 (OCH_3_), 68.88 (CH_2_), 114.23 (2C), 121.54, 123.41, 124.51, 126.10, 126.90, 126.99, 127.90, 128.13, 129.42, 129.76, 130.21, 132.41, 135.32, 136.17, 139.21, 147.13, 149.59, 149.69, 151.65, 161.65, 169.93, 195.97 (C=S). Anal. calcd for C_27_H_21_N_3_O_4_S_2_: C, 62.90; H, 4.11; N, 8.15. Found: C, 62.56; H, 4.20; N, 7.85.

###### 5-{4-[(3-(4-Methoxyphenyl)-4-oxo-3,4-dihydroquinazolin-2-yl)methoxy]-3-methoxybenzylidene}-2-thioxothiazolidin-4-one (**50**)

Yield: 48%, mp 148–150 °C (DMF/H_2_O); ^1^H NMR (DMSO-d_6_, ppm): δ 3.80 (s, 3H, OCH_3_), 3.83 (s, 3H, OCH_3_), 4.81 (s, 2H, CH_2_), 6.95–7.07 (m, 4H, Ar–H), 7.16 (s, 1H, Ar–H), 7.40–7.42 (d, *J* = 8.70, 2H, Ar–H), 7.57–7.61 (m, 2H, Ar–H, azomethine-H), 7.67–7.70 (m, 1H, Ar–H), 7.84–7.89 (m, 1H, Ar–H), 8.14–8.16 (d, *J* = 8.10 Hz, 1H, Ar–H). ^13^C NMR (DMSO-d_6_, ppm): δ 55.85 (OCH_3_), 56.20 (OCH_3_), 68.77 (CH_2_), 114.39, 114.47, 114.85 (2C), 121.54, 123.31, 124.54, 126.92, 126.98, 127.84, 127.98, 128.68, 130.33 (2C), 132.49, 135.25, 147.16, 149.70, 149.87, 152.12, 159.93, 161.88, 169.86, 195.94 (C=S). Anal. calcd for C_27_H_21_N_3_O_5_S_2_: C, 61.00; H, 3.98; N, 7.90. Found: C, 61.10; H, 3.90; N, 8.07.

### Biology

#### Cell culture and assessment of cytotoxicity

The compounds were tested for their cytotoxic activity on a solid tumor cell line, i.e. HT-1080 originating from a fibrosarcoma (American Type Culture Collection; ATCC, Rockville, MD, USA), and on two leukemia cell lines, i.e. the HL-60 human promyelocytic leukemia (European Collection of Animal Cell Cultures; ECACC, Salisbury, UK) and the K-562 human chronic myelogenous leukemia (ATCC). Furthermore, the human skin fibroblast strain AG01523 (Coriell Institute for Medical Research, Camden, NJ, USA) was also used as normal control. Adherent cells were routinely cultured in Dulbecco’s minimal essential medium (DMEM), and leukemia cells in RPMI 1640, in an environment of 5% CO_2_, 85% humidity, and 37 °C. All media were supplemented with penicillin (100 U/ml), streptomycin (100 μg/ml) (media and antibiotics from Biochrom KG, Berlin, Germany), and 10% fetal bovine serum (Life Technologies Europe BV, Thessaloniki, Greece). Adherent cells were subcultured using a trypsin (0.25%; Life Technologies Europe BV)—citrate (0.30%; Sigma, St. Louis, MO, USA) solution. The cytotoxicity assay was performed by a modification of the MTT method [41, 42]. Briefly, the cells were plated in flat-bottomed 96-well microplates at a density of 5000 cells/well, and incubated overnight before the addition of serial dilutions of the test compounds. The cells were incubated with the compounds or the corresponding vehicle (DMSO) concentrations for 3 days. Then, the medium was replaced with MTT (Sigma) in serum-free, phenol-red-free DMEM (1 mg/ml). After incubation for 4 h, the MTT formazan was solubilized in 2-propanol, and the optical density was measured using a FLUOstar Optima (BMG Labtech, Ortenberg, Germany) microplate reader at a wavelength of 550 nm (reference wavelength 660 nm). Doxorubicin hydrochloride (Sigma) was included in the experiments as positive control. The results represent the mean of three independent experiments and are expressed as IC_50_ [42].

#### Western analysis of protein expression

Apoptosis was estimated based on caspase-3 cleavage, as previously described [40]. Briefly, exponentially growing HL-60 cells were incubated with the test molecules at 50 μM for 48 h. Cell lysates were collected in hot sample buffer (62.5 mM Tris, pH 6.8, 6% w/v SDS, 2% v/v glycerol, 5% v/v 2-mercaptoethanol, 0.0125% w/v bromophenol blue, and protease and phosphatase inhibitor cocktails), sonicated for 15 s, clarified by centrifugation and stored at – 800 °C until use. They were separated on 12.5% SDS-PAGE and the proteins were transferred to Polyscreen PVDF membranes (Perkin Elmer, Thessaloniki, Greece). After blocking with 5% (w/v) non-fat dried milk in 10 mM Tris–HCl, pH 7.4, 150 mM NaCl, and 0.05% Tween-20 (TTBS) buffer, membranes were incubated with the appropriate primary antibodies, i.e. rabbit polyclonal anti-caspase-3 (Cell Signaling Technology, Hertfordshire, UK) or mouse monoclonal anti-actin (Neomarkers, Lab Vision Corporation, Fremont, CA, USA). Then, they were washed with TTBS, incubated with either anti-mouse or anti-rabbit horseradish peroxidase-conjugated goat secondary antibody (Sigma), washed again with TTBS and the immunoreactive bands were visualized by chemiluminescence (LumiSensor HRP Substrate Kit, GenScript, Piscataway, NJ, USA) according to the manufacturer’s instructions on a Fujifilm LAS-4000 luminescent image analyzer (Fujifilm Manufacturing, Greenwood, SC, USA).

#### Intracellular reactive oxygen species determination

Intracellular ROS accumulation was studied using a modification of the DCFH-DA method [43]. In particular, HT-1080 cells were plated in black flat-bottomed 96-well microplates at a density of 10,000 cells/well, and left to adhere overnight. Then, they were loaded with 10 μM 2′,7′-dichlorofluorescein diacetate (DCFH-DA) for 1 h, followed by addition of the test molecules at 10 μM. Fluorescence at 520 nm after excitation at 480 nm was measured at various time-points using a FLUOstar Optima microplate reader. The last measurement was taken at 48 h post stimulation. Then, the cells were fixed in 20% methanol, stained with 0.5% w/v crystal violet in 20% methanol, and the wells were washed with deionized water. The stain was solubilized in 10% acetic acid, and the absorbance was measured in the above microplate reader at 550 nm. DCF fluorescence was normalized to the cell number, as assessed indirectly by the crystal violet staining.

## Conclusions

Among the rhodanines reported in the present study, compounds **45**, **43**, **47** and **37** were the most active, especially against leukemia cell lines, exhibiting in vitro cytotoxic activity in the low micromolar range. Structure–activity relationship of the tested compounds revealed that bulky, hydrophobic, and electron-withdrawing substituents at the *para*-position of the quinazolinone 3-phenyl ring enhance cytotoxicity. In addition, methoxy substitution on the central benzene ring was also found to have a positive impact on cytotoxicity. Selectivity against cancer cells as opposed to normal ones was also observed. Mechanistic studies revealed that the most cytotoxic target compounds exhibit pro-apoptotic activity and trigger oxidative stress in cancer cells. In our ongoing research project, further in depth mechanistic investigation as well as molecular modeling studies will be performed to obtain novel therapeutic candidates with improved pharmacological profile.
